# *BRCA1/2* potential founder variants in the Jordanian population: an opportunity for a customized screening panel

**DOI:** 10.1186/s13053-023-00256-2

**Published:** 2023-07-03

**Authors:** Olfat Ahmad, Christian Sutter, Steffen Hirsch, Stefan M. Pfister, Christian P. Schaaf

**Affiliations:** 1 Division of Pediatric Neurooncology, Hopp Children’s Cancer Center (KiTZ), Heidelberg, Germany; 2 Division of Pediatric Neurooncology, German Cancer Research Center (DKFZ), German Cancer Consortium (DKTK), Heidelberg, Germany; 3 Institute of Human Genetics, Heidelberg University, Heidelberg, Germany; 4 University of Oxford, Oxford, UK; 5 King Hussein Cancer Center (KHCC), Amman, Jordan; 6 Department of Pediatric Hematology and Oncology, Heidelberg University Hospital, Heidelberg, Germany

**Keywords:** *BRCA1*, *BRCA2*, Founder variants, Breast cancer, Jordan, Customized screening panel, Population-based screening

## Abstract

A founder variant is a genetic alteration, that is inherited from a common ancestor together with a surrounding chromosomal segment, and is observed at a high frequency in a defined population. This founder effect occurs as a consequence of long-standing inbreeding of isolated populations. For high-risk cancer predisposition genes, such as *BRCA1/2,* the identification of founder variants in a certain population could help designing customized cost-effective cancer screening panels. This advantage has been best utilized in designing a customized breast cancer *BRCA* screening panel for the Ashkenazi Jews (AJ) population, composed of the three *BRCA* founder variants which account for approximately 90% of identified *BRCA* alterations. Indeed, the high prevalence of pathogenic *BRCA1/2* variants among AJ (~ 2%) has additionally contributed to make population-based screening cost-effective in comparison to family-history-based screening. In Jordan there are multiple demographic characteristics supporting the proposal of a founder effect. A high consanguinity rate of ~ 57% in the nineties of the last century and ~ 30% more recently is a prominent factor, in addition to inbreeding which is often practiced by different sub-populations of the country.

This review explains the concept of founder effect, then applies it to analyze published Jordanian *BRCA* variants, and concludes that nine pathogenic (P) and likely pathogenic (LP) *BRCA2* variants together with one pathogenic *BRCA1* variant are potential founder variants. Together they make up 43% and 55% of all identified *BRCA1/2* alterations in the two largest studied cohorts of young patients and high-risk patients respectively. These variants were identified based on being recurrent and either specific to ethnic groups or being novel. In addition, the report highlights the required testing methodologies to validate these findings, and proposes a health economic evaluation model to test cost-effectiveness of a population-based customized *BRCA* screening panel for the Jordanian population. The aim of this report is to highlight the potential utilization of founder variants in establishing customized cancer predisposition services, in order to encourage more population-based genomic studies in Jordan and similar populations.

## Background

### Basic definitions: founder effect, founder population and founder variant

When a new population is founded by individuals who are randomly withdrawn from an ancestral population and then allowed to undergo rapid growth, a so-called “Founder Population” with decreased genetic variation in comparison to the parental population is created [1]. This effect could also be observed in populations which practice inbreeding, and results in enrichment of founder variants within respective populations. The high prevalence of some autosomal recessive disorders within certain founder populations are classic examples of this effect, such as Tay-Sachs disease in Ashkenazi Jews (AJ) [2], Ellis-van Creveld Syndrome in the Amish [3], Cystic Fibrosis in the Hutterites [4] and Congenital Chloride Diarrhea in the Finnish [5]. This feature has allowed founder populations to become valuable sources for studying the prevalent disorders within them. In the field of cancer predisposition, founder populations have also allowed the identification of multiple ethnic-specific pathogenic variants of known autosomal-dominantly inherited disease-causing genes, which has exerted great implications on genetic screening in their respective populations (e.g. *BRCA* screening panel of the AJ [6], the Polish [7, 8] and the Chilean [9]).

### *BRCA* founder variants: benefit of applying customized population-based screening

Breast cancer is the most common cancer worldwide, and it is estimated that up to ~ 15% of all cases are hereditary [10]. *BRCA1/2* pathogenic/likely pathogenic (P/LP) germline variants are the most common underlying genetic causes, and they are known to be associated with high penetrance rates, aggressive pathological phenotypes and earlier age of onset [11]. Since identification of the *BRCA1/2* genes around 30 years ago [12, 13], several other genes related to hereditary breast cancer with variable penetrance rates have been identified (e.g. *TP3*) [14], and genetic testing and counseling have become an integral part of routine clinical practice. Nevertheless, many experts are continuously calling for expanding current testing guidelines both horizontally by including more patients and vertically by expanding the panel of genes tested for [15, 16]. Mary-Claire King, who was the first to describe *BRCA1*, has suggested that genetic screening, of at least *BRCA1/2*, should be offered to every woman at around the age of 30 years, in order to detect healthy carriers prior to cancer onset [17]. Indeed, several groups have calculated that family-history-based screening for *BRCA* deleterious variants could miss around 50% of carriers in comparison to a population-based screening approach [18]. However, a systematic review of economic evaluations of population-based vs. family-history-based *BRCA* testing proved cost-effectiveness only in high and upper-middle income countries and those with prevalent gene mutations, while population-based genetic testing for low-middle income countries was limited by the cost of the testing [19]. AJ are considered the first population who have applied a population-based screening, based on a significantly favorable cost-utility analysis (CUA) of applying a customized *BRCA* screening panel (a discounted incremental cost-effectiveness ratio (ICER) of—£2,079/QALY (quality-adjusted life-years), which is way below the threshold of £20,000/QALY according to the NHS policy in the United Kingdom (UK)) [18]. Besides the income of the country and the cost of testing panel, there are two main factors contributing to making this approach particularly significantly cost-effective for the AJ, which also make it a promising health economic approach for the Jordanian population. First the customized AJscreening panel detecting the three founder *BRCA1/2* variants [6], which make ~ 90% of all possible pathogenic variants among the AJ, and second the high frequency of *BRCA1/2* carriers (~ 2% of the AJ population, as identified by the GCaPPS study) [20].

Similarly, identifying founder *BRCA* variants has also been employed to develop simple affordable testing strategies in other involved populations, such as in Poland, where three variants have been identified to contribute to ~ 90% of all *BRCA* variants [7, 21], and in Chile, where nine founder variants make 78% of all identified *BRCA1/2* variants [9]. Similar to the AJ, both populations are considered relatively homogenous. While the majority of the Polish population are believed to come from the seven European Clan mothers, [22] the founder effect in Chile could be attributed to the dramatic reduction of population due to smallpox and wars with the Spanish creating the scarce founder population of limited genetic variability which inflated during the subsequent 300 years [9].

This report analyzes published Jordanian *BRCA1/2* variants, taking into consideration the historical demographic characteristics of the Jordanian population which could support the founder effect proposal. The report then provides an insight how to employ these findings to develop a customized *BRCA* screening panel for the Jordanian population. Finally, it provides a road-map of further steps needed to prove the founder variants and to conduct a CUA of the customized panel with the aim of facilitating decision making on this regard for the policy makers of the Jordanian health sector.

## Main text

### Breast cancer care in Jordan

Similar to the global situation, breast cancer is the most common malignancy in Jordan accounting for 20.6% of cancers in Jordanians of both sexes and 39.4% of cancers among Jordanian women, with increasing figures by 69% during the past decade [23, 24]. Around 60% of diagnosed Jordanian women are treated at King Hussein Cancer center (KHCC). On the other hand, Al- Bashir Hospital is the largest hospital of the public sector treating a significant share of the remaining 40% of cases. According to limited published data, almost 10–15% of breast cancer occurrences are inherited with the most commonly identified genetic alterations in *BRCA1/2*. The results of *BRCA1/2* genetic testing from Jordan have been published between the years 2018 and 2021 [23, 25–28], in addition to an old report of a smaller cohort dating back to 2004 [29]. Reports are only available from the two major mentioned centers. Since P/LP mutations of *BRCA1/2* genes are associated with early onset breast cancer and/or high-risk features (HR), this review analyzes the results of the two largest published cohorts from KHCC including 616 young female patients (i.e. younger than 40 years) [26] and 500 patients with high-risk (HR) features (median age = 39 years) [28], together with the largest published cohort of 192 female patients with HR features from Al-Bashir hospital (mean age = 43.6 years) [23].

### Potential Jordanian *BRCA1/2* founder variants

Eighty (12.2%) patients of the first cohort, seventy-two (13%) of the second and twenty-nine (14.5%) of the third had a P/LP variant of *BRCA1/2* [23, 26, 28]. The prevalence of P/LP *BRCA1/2* gene mutations among the specific subgroup of triple negative (TN) disease was 33.9%, and it was 60% among patients with both TN disease and a family history of breast cancer as published by KHCC group [30]. There are sixteen recurrent variants (i.e. occurring in two or more patients) which were identified in these two cohorts, as summarized in Tables 1 and 2. Further analysis of these variants leads to the proposal that ten could be founder variants, including nine *BRCA2* variants and a single *BRCA1* variant. Significantly, this group composes 43%, 55% of all identified *BRCA1/2* variants among the two studied cohorts from KHCC, while the percentage was not easy to calculate from the third cohort, as the paper has only provided the results of 16 of the 29 reported patients with P/LP variants of *BRCA1/2* (table 3 of the original paper [23]). In addition to the observation that all of the identified ten variants are recurrent, four are Palestinian-specific, four are specific to European/Caucasian ethnicity, and two are novel. In addition, one of these novel variants co-occurred with another variant in several patients suggesting the possibility of them being inherited as part of a mutual allele (Table 1). *BRCA2* c.2254_2257del p.(Asp752Phefs*19) is the most common variant, accounting for 11/55 (20%) *BRCA2* variant findings in the cohort of young patients, 8/48 (16.7%) in the second cohort and 2/8 (25%) of the third cohort. This variant has only been reported previously in a Palestinian patient [31]. Furthermore, five (six) of the patients, additionally carry *BRCA2* c.5351dup p.(Asn1784Lysfs*3) in the first (second) cohort (Table 1) [27]. Both variants are located in exon 11, and their co-occurrence has not been reported previously [32–37], suggesting that they may have been both inherited as part of a shared founder allele from a common Jordanian ancestor. It could be argued here, that only the first variant is a *bona fide* pathogenic one, as it may omit the effect of the second downstream variant by its truncating effect on the protein or by nonsense mediated RNA decay. The other novel variant was *BRCA2* c.4222_4223del p.(Gln1408Argfs*5) occurring in 2/55 cases among young patients [26]. The fourth potential founder variant was a *BRCA2* exon 5–11 duplication, which occurred in 8/55 [26] and 5/48 patients [28]. This variant has been previously reported for Christian Palestinians exclusively, with a frequency of 5/33 [38]. The other two identified Palestinian-specific variants were: *BRCA2* c.6685G > T p.(Glu2229Ter), and *BRCA2* c.6627_6634del p.(Ile2209Metfs*13). On the other hand, only one of the identified recurrent *BRCA1* variants seems to be ethnic-specific, which is *BRCA1* c.5123C > A p.(Ala1708Glu), that was identified in 2/25 [26] and 2/24 patients [28]. This variant is a known Mediterranean founder variant, and has been described in the Spanish [39], Sephardic Jews [40] and Hispanics [41] (Table 2). In order to understand how Palestinian and Mediterranean founder variants could be mutual with the Jordanian population, a few historical characteristics of the Jordanian population need to be understood.

**Table 1:** Recurrent pathogenic and likely pathogenic *BRCA2* variants of Jordanian patients with breast cancer

**Gene**	**Exon**	**Variant nomenclature (as appeared in reference report)**	**Variant nomenclature acc. to HGVS designation**^b^	**Variant type**	**Variant class**	**Frequency in the 2 largest published Jordanian cohorts, compared to total numbers (N) of identified *BRCA2* variants in each report**			**Ethnicities/Nationalities where variants have been previously reported**
						**A cohort of 616 young patients** [26] **from KHCC** **(*N* = 55)**	**A cohort of 500 HR patients** [28] **from KHCC** **(*N* = 48)**	**A cohort of 192 HR patients from MoH** [23] **(*N* = 8)**	
***BRCA2***	11	2482del4 [27], c.2254_2257del (p.Asp752Phefs) [23, 25, 26, 28]	c.2254_2257del p.(Asp752Phefs^a^19)	fs	P*	11	8	2	Palestinian [33, 35]
***BRCA2***	11	c.2254_2257del (p.Asp752Phefs) co-occurring with c.5351dup (p.Asn1784Lysfs) [25, 26, 28]	c.2254_2257del p.(Asp752Phefs^a^19) and c.5351dup p.(Asn1784Lysfs^a^3) NM_000059.4:c. 2254_2257del(;) 5351dup	fs	P*	5	6	0	Novel [32–37]
***BRCA2***	5–11	Exon 5–11 duplication [25, 26, 28], dup exons 5–11(5’)[27]	Exon 5–11 duplication; c.(425 + 1_426-1)_(6841 + 1_6842-1)dup p?	large dup	P	8	5	0	Christian Palestinians [33, 35, 38]
***BRCA2***	11	c.6685G > T (p.Glu2229Ter) [25, 26, 28] 6913G > T/E2229X [27]	c.6685G > T p.(Glu2229^a^)	ns	P*	3	3	0	Palestinian [42], and Jordanian [43]
***BRCA2***	10	1461insA [27] c.1233dup (p.Pro412Thrfs) [25, 26, 28]	c.1233dup p.(Pro412Thrfs^a^9)	fs	P*	5	3	0	European [33, 35]
***BRCA2***	Intron 24	c.9257-1G > A [25, 26, 28] IVS24-1 G > A [27]	c.9257-1G > A p.?	sp	P*	2	2	0	Western European [33, 35]
***BRCA2***	11	c.6486_6489del p.Lys2162Asnfs [26, 28]	c.6486_6489del p.(Lys2162Asnfs^a^5)	fs	P*	2	2	0	Western European [33, 35]
***BRCA2***	11	c.4222_4223del (p.Gln1408Argfs^a^5) [26, 28]	c.4222_4223del p.(Gln1408Argfs^a^5)	fs	LP	2	0	0	Novel [32–37]
***BRCA2***	11	c.6627_6634del (p.Ile2209Metfs) [26, 28]	c.6627_6634del p.(Ile2209Metfs^a^13)	fs	P*	2	0	0	Jordanian [43, 44], Palestinians, [45] and non-Jewish Israeli [46]
***BRCA2***	22	c.8878C > T (p.Gln2960Ter) [25, 26, 28]	c.8878C > T p.(Gln2960^a^)	ns	P*	2	0	0	Multiple Ethnicities [35]

*Abbreviations*: *KHCC* King Hussein Cancer Center, *MoH* Ministry of health, *HR* High risk, *R* Reference number, *N* total number of identified *BRCA2* events in a certain reference, *fs* frameshift, *ns* nonsense, *sp* splice variant, *large dup* large duplication, *P* pathogenic, *LP* likely pathogenic

^a^ClinVar classification

^b^cDNA refseq: NM_000059.4 (MANE Select)

**Table 2:** Recurrent pathogenic and likely pathogenic *BRCA1* variants of Jordanian patients with breast cancer

**Gene**	**Exon**	**Variant nomenclature (as appeared in reference report)**	**Variant nomenclature acc. to HGVS designation**^b^	**Variant type**	**Variant class**	**Frequency in the largest 2 published Jordanian cohorts, as compared to total number (N) of identified *BRCA1* events in each report**			**Ethnicities/Nationalities where variants have been previously reported in the literature**
						**A cohort of 616 young patients from KHCC** [26] **(*N* = 25)**	**A cohort of 500 HR patients from KHCC** [28] **(*N* = 24)**	**A cohort of 192 HR patients from MoH** [23] **(*N* = 8)**	
***BRCA1***	2	c.66dup (p.Glu23Argfs) [26] c.66dup (p.Glu23Arg)^c^ [28]	c.66dup p.(Glu23Argfs^a^18)	dup	P^a^	1	5	0	Multiple Ethnicities [35, 44]
***BRCA1***	12	c.4117G > T (p.Glu1373Ter) [25, 26, 28] E1373X [27]	c.4117G > T p.(Glu1373Ter)	ns	P^a^	4	3	0	Multiple Ethnicities [35, 44]
***BRCA1***	Intron 17	c.5074 + 3A > G/ IVS17 + 3A > G [25, 26, 28]	c.5074 + 3A > G p.?	sp	P/LP^a^	3	3	0	Multiple Ethnicities [35, 44]
***BRCA1***	3	c.121C > T (p.His41Tyr) [25, 26, 28]	c.121C > T p.(His41Tyr)	ms	P/LP^a^	1	2	2	Multiple Ethnicities [35, 44]
***BRCA1***	18	c.5123C > A (p.Ala1708Glu) [25, 26, 28]	c.5123C > A p.(Ala1708Glu)	ms	P^a^	2	2	0	Founder variant of Spanish [39], Sephardic Jews [40] and Hispanics [41]
***BRCA1***	11	c.4065_4068del (p.Asn1355Lysfs) [25, 26, 28]	c.4065_4068del p.(Asn1355Lysfs^a^10)	fs	P^a^	2	2	2	Multiple Ethnicities [35, 44]

*Abbreviations*: *KHCC* King Hussein Cancer Center, *MoH* Ministry of health, *HR* High risk, *R* Reference number, *N* total number of identified *BRCA1* events in a certain reference, *dup* duplication, *fs* frameshift, *ns* nonsense, *ms* missense, *sp* splice variant, *P* pathogenic, *LP* likely pathogenic

^a^ClinVar classification

^b^cDNA refseq: NM_007294.4 (MANE Select)

^c^this is how the nomenclature of the protein appeared in reference 28, however, we believe it was a typing mistake, since this is a fs variant, as also was mentioned in reference 26 from the same group of Abdel-Razeq et al

### Understanding the historical demographic characteristics of the Jordanian population that could have created a founder effect

**Fig. 1:**
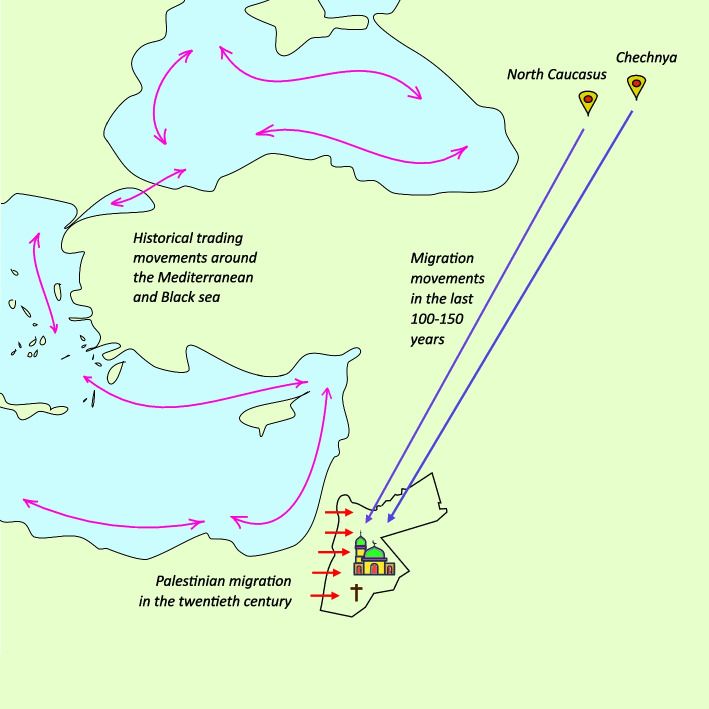
Mapping historical migratory movements to Jordan from Chechnya and the North Caucasus, which contribute to the two big ethnic minorities of the country. The figure also illustrates the third minority of Christian Arabs, together with the relationship between Jordanians and Palestinians who fled to the country during Palestinian-Israeli wars and currently make about half of the population. In addition, the interaction of populations around the Mediterranean and the Black sea are illustrated

Jordan is a home of about 10.8 million inhabitants, with a majority of Arabs (98%) [47]. There are two major well-mixed communal groups in the country: Jordanians who originally come from Jordan, which is the land laying East to Jordan River, and Jordanians of Palestinian origin, who mainly fled to the country as a consequence of Palestinian-Israeli wars in 1948 and 1967 [48]. Secondly, while 4% of Arabs in the country are Christians [47], Circassians and Chechens represent the biggest two ethnic minorities in Jordan, with estimated percentages of ~ 1% [49] and ~ 0.1% [50] of the Jordanian population respectively (Fig. 1). Inbreeding is a common practice of the latter three subpopulations, and disparity in the distribution of mtDNA haplotype frequencies among Jordanian Arabs, Circassians and Chechens was demonstrated by Al-Eitan et al. in 2019 [51]. In a similar vein, up to twice as high crude rates of breast cancer in Circassians and Chechens in comparison to Arab Jordanians were reported in 2013, with (95%CI) rate ratios of 2.1 (1.48, 2.72) and 1.81 (1.16, 2.85), respectively [52]. Secondly, mutual Mediterranean founder variants, could be attributed to historical migratory movements, trade activities, and other populations’ interactions that used to occur along the Mediterranean and the Black sea (Fig. 1) [53], which could explain the recurrence of the three European/Caucasian *BRCA2* variants and the known Mediterranean *BRCA1* founder variant. Thirdly, even though Al-Eitan’s study did not include analysis in respect to religion, the fact that the recurrent *BRCA2* exon 5–11 duplication has only been previously reported among Christian Palestinians [38], together with the tendency of this subpopulation to get married to Jordanians or Palestinians of the same religion, most likely makes this variant a potential founder variant for the Christian Jordanian-Palestinian population. Finally, consanguinity is a very common practice all over the country. In 1990, it was estimated that 57% of marriages in Jordan were consanguineous [54]. Despite the downward trend of this figure over the years, it still contributes to 30% of marriages in the country [54], which supports the proposal of potential founder effect in the general Jordanian population, which could be indeed thought of as a whole population descending from a few families whose offspring had been breeding with their cousins over the ages.

### A roadmap for developing a customized panel for the Jordanian population

Development of a customized screening panel for the Jordanian population would start with proving that the proposed variants are true founder variants. There are two types of association studies used for this purpose: Haplotype Analysis and Identity By Descent (IBD) studies, both studies help identify the longer shared DNA segments among affected patients who harbor the same potential founder variants, or in other words help to prove that the variants have been inherited as part of a mutual founder allele coming from a common ancestor [55]. Based on the size of the shared allele, several computational tools could be applied to predict the variant age [56], which could then be correlated to potential historical events contributing to the creation of the founder effect. Having those founder variants confirmed would allow the development of a customized test for the Jordanian population, offering ‘targeted screening’ for the common ten *BRCA1/2* variants for lesser costs and faster turnaround times than conventional NGS panel sequencing of the *BRCA* genes. The cost of this customized test needs to be well-identified by communication with candidate provider companies before proceeding with conducting the health economic evaluation of a population-based screening vs. family-history-based screening. In addition, various probabilities and costs should be actively identified by conducting several further dedicated studies. A suggested model for such a CUA could be learnt from the AJ experience, particularly the analysis conducted by Manchanda et al. [18]. It can be observed from this model, that in order to proceed with the analysis, the frequency of *BRCA1/2* mutations among the Jordanian population needs to be identified. The GCaPPS study of AJ has calculated the prevalence by testing a random sample of 1034 healthy volunteers of the AJ of both genders [20]. Other probabilities needed for the analysis would include the likelihood of the uptake of the screening panel by Jordanian population once made available, and the likelihood of an identified carrier to undergo risk-reducing mastectomy (RRM), with or without risk-reducing salpingo-oophorectomy (RRSO), which could be estimated by conducting a Knowledge, Attitude, and Practice (KAP) survey. In the CUA conducted by Manchanda et al., a test uptake of 71% of the general population was assumed as estimated by the GCaPPS study, together with a 52% probability of undergoing RRM and a 55% probability of undergoing RRSO, based on previously conducted KAP surveys [57, 58]. In addition, several studies have been conducted to study the factors affecting the attitude of AJ toward uptake of prophylactic surgeries, which might help predict the attitude of other populations. Next step of the analysis would be estimating QALYs of each health state in the model by multiplying their utility weights by the survival in life years, where utility weights range from 1 for a perfect health and 0 for death. Needed utility weights are available in the literature [59–62]. Finally, the costs of breast cancer treatment and ovarian cancer treatment need to be actively assessed by calculating the costs of different stages of both diseases in Jordan. Having that all done would allow ICER calculation to decide on the cost utility of the intended customized screening panel.

## Conclusion

To conclude, understanding the concept of a founder mutation has allowed implementing cost-effective policies for genetic testing and screening for carriers in respective populations. Population-based screening for *BRCA* carriers has been suggested by several experts in the field and has been shown to be cost-effective for middle and high-income countries. Jordan, as an upper-middle-income country, where 43–55% of all identified *BRCA* events are potential founder events, has a real opportunity to benefit of a customized *BRCA* screening panel to detect healthy carriers prior to costly disease treatment. Indeed, adopting such an early detection approach would certainly decrease the current estimated incidence of late diagnoses of breast cancer in the country, which makes ~ 12% of new cases (double the rate of developed countries) [15]. This is the first report highlighting the preponderance of *BRCA1/2* founder variants in Jordan. It is also the first report explaining how the historical demographic characteristics of the country could have created a founder effect, which might have similarly affected other genes in Jordan and in neighboring countries of similar historical and demographic features. On the other hand, a few limitations of this study need to be addressed. The first is the frequencies of potential founder *BRCA* variants, which have been estimated from the largest two published cohorts from KHCC and the available cohort from Al-Bashir hospital. A more accurate calculation by combining the data of all tested patients at KHCC would help calculate frequencies more precisely. Secondly, a threshold of two or more patients is used in this report to define recurrent variants. No clinical data have been available to examine if the identified variants are coming from different families, which would have strengthened our proposal, especially for the variants of lowest frequencies. Nevertheless, six out of the ten identified variants would still meet an increased threshold of three or more patients, and four different variants occur in five or more patients. Lastly, no data have been available to support the proposal that the two co-occurring variants on exon 11 of *BRCA2* gene are in cis orientation (i.e. on the same allele). Thirdly, as mentioned in the manuscript, it was difficult to calculate the prevalence of the shortlisted variants among the cohort from Al-Bashir hospital due to limited published data. Nevertheless, it was good to identify *BRCA2* c.2254_2257del as the most common variant at KHCC and Al-Bashir hospital, despite the small numbers of the latter cohort. Finally, it has to be clearly stated that this report focuses on a population-based screening approach aiming at early detection of healthy carriers. Other non-*BRCA1/2* genes associated with breast and/or ovarian cancer (in less than 6% of hereditary cases [14]), are targeted by NGS-based multigene panel diagnostics adopted for affected families, which is not the focus of this report.

## Data Availability

Not applicable.

## Abbreviations

AJ: Ashkenazi Jews
P: Pathogenic
LP: Likely pathogenic
CUA: Cost-utility analysis
ICER: Incremental cost-effectiveness ratio
QALYs: Quality-adjusted life years
UK: United Kingdom
HR: High-risk
TN: Triple Negative
IBD: Identity By Descent
RRM: Risk-reducing mastectomy
RRSO: Risk-reducing salpingo-oophorectomy
KAP: Knowledge, Attitude, and Practice
